# Scorpion Toxin, BmP01, Induces Pain by Targeting TRPV1 Channel

**DOI:** 10.3390/toxins7093671

**Published:** 2015-09-14

**Authors:** Md Abdul Hakim, Wenbin Jiang, Lei Luo, Bowen Li, Shilong Yang, Yuzhu Song, Ren Lai

**Affiliations:** 1 Key Laboratory of Animal Models and Human Disease Mechanisms, Kunming Institute of Zoology, Chinese Academy of Sciences, Kunming 650223, China; E-Mails: hakeem.geb.ru@gmail.com (M.A.H.); aleismile@126.com (L.L.); 13700675996@126.com (B.L.); 2 Graduate University of Chinese Academy of Sciences, Beijing 100049, China; 3 Faculty of Life Science and Technology, Kunming University of Science and Technology, Kunming 650223, China; E-Mail: sewend@163.com; 4 Joint Laboratory of Natural Peptide, University of Science and Technology of China and Kunming Institute of Zoology, Chinese Academy of Sciences, Kunming 650223, China

**Keywords:** scorpion *Mesobuthus martensii*, peptide toxin, BmP01, Kv channels, TRPV1, pain

## Abstract

The intense pain induced by scorpion sting is a frequent clinical manifestation. To date, there is no established protocol with significant efficacy to alleviate the pain induced by scorpion envenomation. One of the important reasons is that, little information on pain-inducing compound from scorpion venoms is available. Here, a pain-inducing peptide (BmP01) has been identified and characterized from the venoms of scorpion (*Mesobuthus martensii*). In an animal model, intraplantar injection of BmP01 in mouse hind paw showed significant acute pain in wild type (WT) mice but not in TRPV1 knock-out (TRPV1 KO) mice during 30 min recording. BmP01 evoked currents in WT dorsal root ganglion (DRG) neurons but had no effect on DRG neurons of TRPV1 KO mice. Furthermore, BmP01 evoked currents on TRPV1-expressed HEK293T cells, but not on HEK293T cells without TRPV1. These results suggest that (1) BmP01 is one of the pain-inducing agents in scorpion venoms; and (2) BmP01 induces pain by acting on TRPV1. To our knowledge, this is the first report about a scorpion toxin that produces pain by targeting TRPV1. Identification of a pain-inducing compound may facilitate treating pain induced by scorpion envenomation.

## 1. Introduction

The evolutionary history of the scorpions was begun around 425–450 million years ago, in the Middle Silurian, and these animals are considered as “living fossils” [1]. Scorpions are morphologically conservative organisms and approximately 2069 species are recognized and classified into 15 families [2]. Animal venoms, including venom from scorpions, have been proven as an extremely diverse bioactive pool for predation and defense [3,4,5,6]. Scorpion stings are dangerous to humans, which can induce different levels of toxicity and causes lethal consequences in certain cases [7]. Many neurotoxins from scorpion venom have been explored by genome sequencing [3] as well as transcriptomic and proteomic analysis [8,9]. Peptide toxins from scorpion venoms specifically target ion channels (Na^+^, Cl^−^, K^+^ and Ca^2+^) and other cell membrane receptors, suggesting the functional diversity of scorpion toxins [10,11,12,13]. In the field of pain, peptide toxins have provided important evidence for basic research and clinical applications [14]. Due to the importance of peptide toxins from scorpion, the nature of scorpion venoms should be more concerned and thoroughly investigated. Unfortunately, up to this moment, few scorpion venoms and scorpion toxins, which activate voltage-gated sodium channels, have been reported to produce pain [15,16,17,18,19,20,21,22]. However, the molecular mechanism of pain-producing compounds from scorpion venoms is far from clearly understood.

Bites and stings from venomous animals can induce pain as well as inflammation as a part of their defensive strategy to protect themselves from predators and competitors [23,24]. There are several ion channels expressed in sensory neurons, such as transient receptor potential (TRP) channels, acid-sensing ion channels (ASICs) and voltage-gated ion channels (VGICs), which have been generally considered as principal players in pain signaling pathway [25,26,27,28,29]. Among those channels, the capsaicin (vanilloid) receptor TRPV1 is a heat-activated cation channel that can be stimulated by inflammatory factors and leads to pain symptoms [30]. TRPV1 is the key receptor to detect noxious heat and thermal hypersensitivity. Furthermore, TRPV1 enjoys a variety of pharmacology, including small molecular agonists and antagonists, as well as larger peptide toxins [31]. These molecular compounds evoke tingling and burning pain by activating TRPV1 on sensory nerve endings [32]. The characteristics suggest that TRPV1 is an ideal target for producing pain and inflammatory effects by poisonous animal’s envenomation. Peptide toxins such as VaTx and DkTx from spider venoms have been proven as potent agonists on TRPV1 channel [31,32] and provided new tools for addressing questions related to the structure and gating mechanisms of TRPV1. According to these studies, TRPV1 seems to be a general target for the defense-use peptide toxins of poisonous animals [33].

Although scorpion stings are known to produce pain, inadequate knowledge is established to reveal the sophisticated biochemical strategy of pain-producing molecules in scorpion venom. BmP01 is a pain-inducing peptide from the Scorpion *Mesobuthus martensii*, a kind of aggressive predator that is widely distributed in eastern Asian countries, including Mongolia, Korea and China. In a previous report, BmP01was purified from crude venom [34] and its cDNA sequence was cloned from the cDNA library of the scorpion *Mesobuthus martensii* [35]. The solution-structure of BmP01 was determined and reported that there are three disulfide bridges (1–4, 2–5 and 3–6) containing the α-helix and β-sheet [36]. By evaluating Kv channel-blocking activity, BmP01 inhibited both rat Kv1.1 and human Kv1.3 [9]. Outward K^+^ currents in rat hippocampal neurons can also be slightly inhibited by BmP01 [36]. Although inhibitors of Kv channels may lead excitation of sensory neurons *in vivo*, severe painful behavior by Kv channel blockage is rarely reported.

We have isolated and characterized BmP01 from the venoms of scorpion *Mesobuthus martensii* and investigated its biological activities in mouse model. In this study, we used a combination of animal behavior tests and electrophysiology to elucidate the molecular mechanism and chemical strategy of BmP01-induced burning pain. Moreover, we used kaliotoxin, a potent inhibitor of Kv1.1 and Kv1.3 [37,38], to determine that the inhibition of Kv1.1 and Kv1.3 could not induce pain behavior in mice model. Here, we report our results on BmP01 induced pain by activating TRPV1 channel.

## 2. Results

### 2.1. Hyrdrophobic Peptide Induces Pain in Mouse Model in Vivo

**Figure 1:**
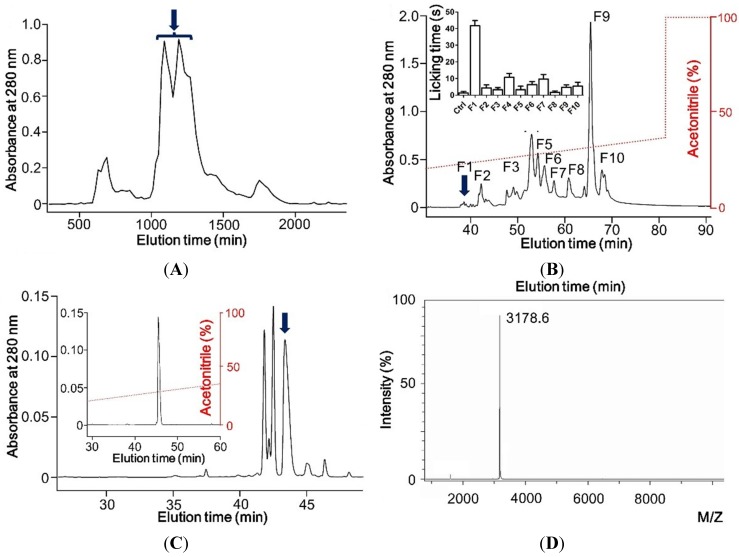
Purification of BmP01 from venom of the scorpion *Mesobuthus martensii*. (**A**) Venom of scorpion was fractionated using Sephadex G-50 gel filtration; (**B**) The peak eluting at 1000–1403 min (indicated by an arrow) was further fractionated on a C_18_ RP-HPLC column. Ten fractions obtained were screened for pain behavioral study in mice. F1 fraction eluting at 38 min (indicated by an arrow) showed the pain inducing activity (inserted panel, *n* = 10); (**C**) F1 was fully purified on an analytical C_18_ RP-HPLC column with a retention gradient of ~35% acetonitrile; (**D**) Molecular weight of the purified peptide was determined to be 3178.6 Da by MALDI-TOF analysis.

In order to explore pain-producing peptides from scorpion venom, we initially isolated and applied the crude venom to Sephadex G-50 (Pharmacia Fine Chemicals, Uppsala, Sweden) column for purification. The crude venom was separated into several fractions by monitoring under ultraviolet at 280 nm (Figure 1A). Among these protein fractions, the fraction containing two peaks marked by arrow was then applied to the C_18_ RP-HPLC (Waters, Milford, CT, USA) column for further purification (RP-HPLC; Gemini C_18_ column, 5 μm particle size, 110 Å pore size, 250 × 4.6 mm). After separation of the fraction, ten fraction components (F1–F10) obtained were screened to investigate the pain behavior by observing paw licking duration in mouse model (Figure 1B). F1, the component (pointed by blue down arrow) having desired pain-producing activity was finally purified using analytical RP-HPLC on a C_18_ column with a retention gradient of ~35% acetonitrile (Figure 1C). The molecular weight of the purified peptide was 3178.6 Da, determined by matrix-assisted laser desorption/ionization time-of-flight (MALDI-TOF) mass spectrometry (Bruker Daltonik GmbH, Leipzig, Germany) (Figure 1D).

### 2.2. Sequence of Pain Inducing Toxin, BmP01

**Figure 2:**
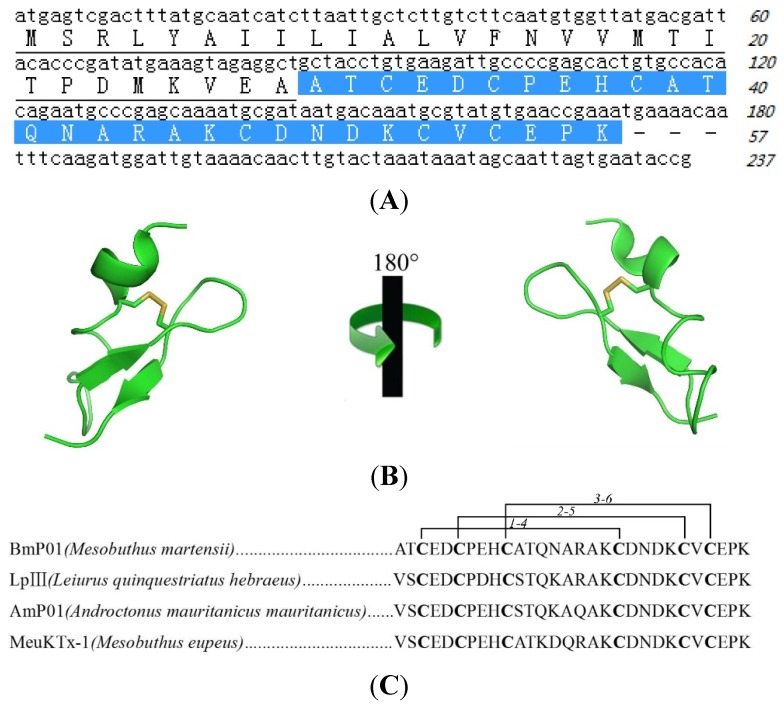
The sequence and structure of BmP01. (**A**) Sequence of cDNA encoding BmP01, the signal peptide (underlined) is of 28 amino acids in length, and the mature peptide consists of 29 amino acids, designated with white text on a blue background; (**B**) The 3D-structure of BmP01 is provided from Protein Data Bank (Protein Data Bank code 1WM7); (**C**) Alignment of purified BmP01 with three previously reported peptides from scorpion. They contain the same number of amino acids and disulfide bonds.

The partial *N*-terminal sequence, determined by automatic Edman degradation, ATCEDCPEHCATQNARAK was used for designing degenerate primers (Table 1) in order to clone the signal and mature peptide sequence from cDNA library. By cloning the gene from venom-gland cDNA library, the complete transcript of BmP01 was identified, revealing that the toxin is translated as a larger precursor of 57 amino acids in length and the mature peptide is yielded during post-translational modification by cleaving off a signal peptide consisting 28 amino acids (Figure 2A). In Figure 2A, the full cDNA and amino acid sequence of this peptide suggest that the peptide we purified is BmP01, which is confirmed by NCBI protein blast. BmP01, an ICK motif toxin with alpha helix and beta sheet, contains three di-sulfide bridges (Figure 2B). The alignment of the mature peptide sequence of BmP01 showed some homologues from other scorpion venoms (Figure 2C).

**Table 1:** The primers used for cDNA cloning of BmP01.

**For Mature Peptide Cloning**
Primer 1 5′-AAGCAGTGGTATCAACGCAGAGTACGCGGG-3′
Primer 2 5′-GCNACNTGYG ARGAY-3′ (N = A/T/G/C; Y = C/T; R = A/G)
**For signal peptide cloning**
Primer 3 5-AAGCAGTGGTATCAACGCAGAGT-3
Primer 4 5′-TTTCGGTTCACATAC-3′

### 2.3. BmP01 Inhibits mKv1.3 but not mKv1.1 Channel

**Figure 3:**
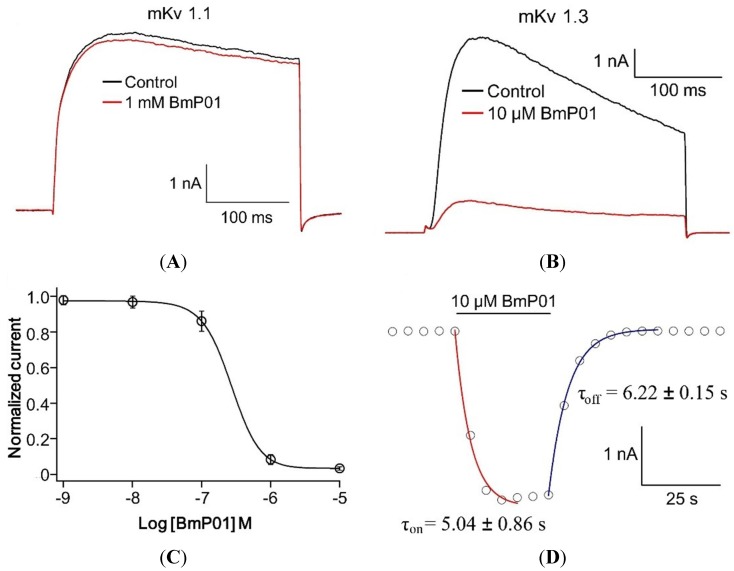
Effects of BmP01 on mouse Kv channels. (**A**) 1 mM BmP01 did not inhibit the mKv1.1 currents; (**B**) 10 μM of BmP01 inhibited the currents on mKv1.3; (**C**) the concentration–response relationship of BmP01 against mKv1.3 yielded an EC_50_ of 269.15 ± 12.69 nM (*n* = 10); and (**D**) on-rate and off-rate of BmP01 interacting with mKv1.1.

Since the peptide, BmP01, from *Buthus martensii*, has been previously reported as rat Kv1.1 and human Kv1.3 channel blocker, we firstly tested the function of native BmP01 on mKv1.1 and mKv1.3 channels. In electrophysiology, application of 10 µM of BmP01 inhibited mKv1.3 currents (Figure 3B), while 1 mM of BmP01 showed no significant effect on mKv1.1 (Figure 3A). To determine the potency of the toxin on mKv1.3, we performed further experiment to measure the concentration—response relationship. The data dots fitted by Hill equation yielded an EC_50_ of 269.15 ± 12.69 nM (Figure 3C). To establish on-rate and off-rate of toxin binding, current amplitudes were fitted by a single-exponential function. In the presence of 10 µM BmP01, the time constants of inhibition and washing were (5.04 ± 0.86 s) and (6.22 ± 0.15 s), respectively (Figure 3D). These findings suggested that the high affinity might be caused by the rapid onset of the BmP01. Assumingly, there may have evolutionary changes of ion channels in murine, which make BmP01 to exhibit inhibitory activity on rKv1.1 but not on mKv1.1. According to these findings in this work, the pain-producing mechanism of BmP01 still remains un-clear; hence, the following experiments were designed and carried out.

### 2.4. BmP01 Induces Pain in WT but not in Trpv1^−/−^ Mice

**Figure 4:**
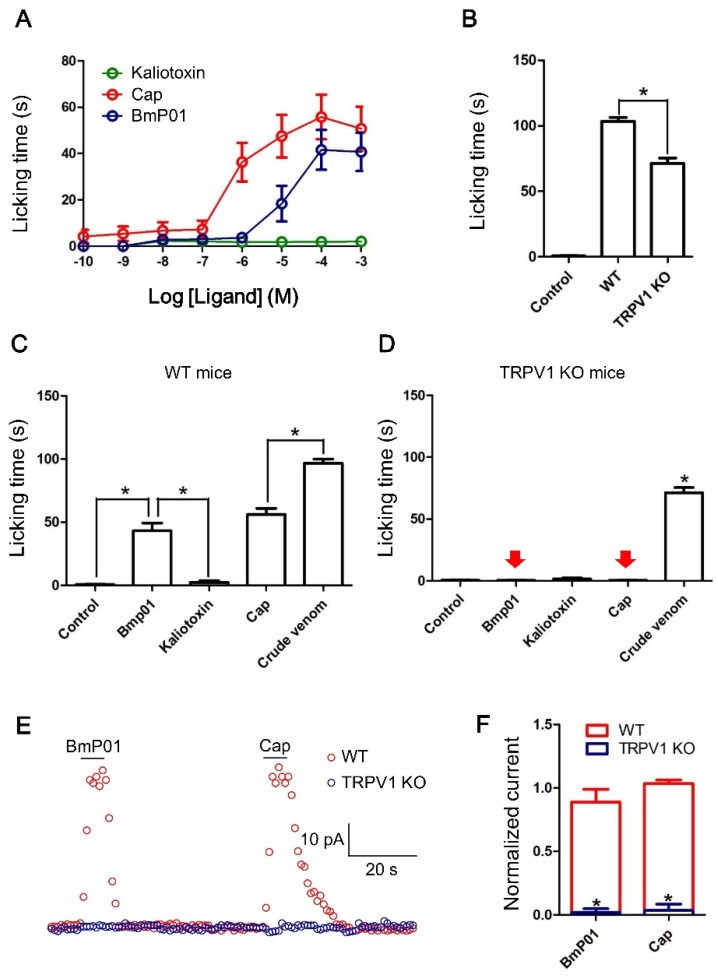
Mean duration (±S.E.) of paw licking and electrophysiology on DRG neurons. (**A**) Different doses of BmP01 along with capsaicin and kaliotoxin were injected into WT mice. Kaliotoxin showed no significant pain behavior, whereas application of 500 µM BmP01 showed acute pain behavior similar to capsaicin; (**B**) Ten microliters (25 ng/μL) Crude venom injected into WT and TRPV1 KO mice showed the significant difference of the pain behavior between WT and TRPV1 KO mice; (**C**) Ten microlites saline (control), 500 μM of BmP01, kaliotoxin, capsaicin and 10 μL (25 ng/μL) crude venom were injected into the paw of WT mice. BmP01 and capsaicin induced pain in WT mice. Kaliotoxin was unable to induce pain whereas crude venom induces severe pain; (**D**) Ten microliters saline (control), 500 μM of BmP01, kaliotoxin, capsaicin and 10 μL (25 ng/μL) crude venom were injected into the paw of TRPV1 KO mice. Similar to capsaicin, BmP01 did not induce pain, while only crude venom induced pain; (**E**) Both BmP01 (300 μM) and capsaicin (10 μM) evoked the currents on DRG sensory neurons of WT mice. Contrastingly, in DRG neurons from TRPV1 KO mouse, BmP01 as well as capsaicin did not evoke currents; (**F**) Normalized currents evoked by BmP01 and capsaicin on DRG neurons of WT and TRPV1 KO mice. For both BmP01 and capsaicin, current traced is significantly lower in TRPV1 KO mice than in WT mice. * *p* < 0.01, *n* = 10.

We investigated the dose-response of kaliotoxin, capsaicin and BmP01 for pain behavior in WT mice. Capsaicin and BmP01 induced acute pain in a dose dependent manner, whereas well known potassium channel inhibitor kaliotoxin (a potent inhibitor of Kv1.1 and Kv1.3) did not induce pain (Figure 4A). In order to investigate whether TRPV1 is one of the targets of pain inducing toxins from scorpion venom, the effect of crude venom was tested in WT and TRPV1 KO mice. Crude venom (25 ng/µL) was injected into the WT and TRPV1 KO mice and it was noticed that there was a significant difference of pain behavior between WT and TRPV1 KO mice (Figure 4B). To test whether BmP01 produces the pain by going through the TRPV1 pathway, 10 μL BmP01 (500 µM) along with capsaicin (500 µM), kaliotoxin (500 µM) and crude venom (25 ng/µL) were tested to check the pain behavior in WT and TRPV1 KO mice. The same volume of saline was injected for control. The duration of licking/biting represented in bar graph showed that BmP01 and capsaicin induced pain in WT mice (Figure 4C). Whereas, surprisingly, similar to capsaicin, BmP01 lost function to induce pain in TRPV1 KO mice (Figure 4D). These findings suggest that BmP01 may play a role to induce pain in the similar way with capsaicin by targeting TRPV1 channels.

### 2.5. BmP01 Evokes Currents on Sensory Neurons in WT but not in Trpv1^−/−^ Mice

Up to this point, although the BmP01 seems to be involved with TRPV1 for inducing pain, more evidence is required to support this hypothesis. Afterward, we tested BmP01 on DRG neurons (20 µm ≤ cell ≤ 35 µm) in WT and TRPV1 KO mice whether it evoked the currents. Interestingly, it was markedly observed that 300 µM BmP01 as well as 10 µM capsaicin effectively evoked the currents in DRG neurons of WT but not TRPV1 KO mice (Figure 4E) and the currents evoked by BmP01 and capsaicin were found significantly higher in WT than TRPV1 KO mice (Figure 4F). These results triggered our interest to investigate whether BmP01-induced currents in neurons were originated from the same target of capsaicin.

### 2.6. BmP01 Peptide Targets mTRPV1 Channel to Induce Pain

**Figure 5:**
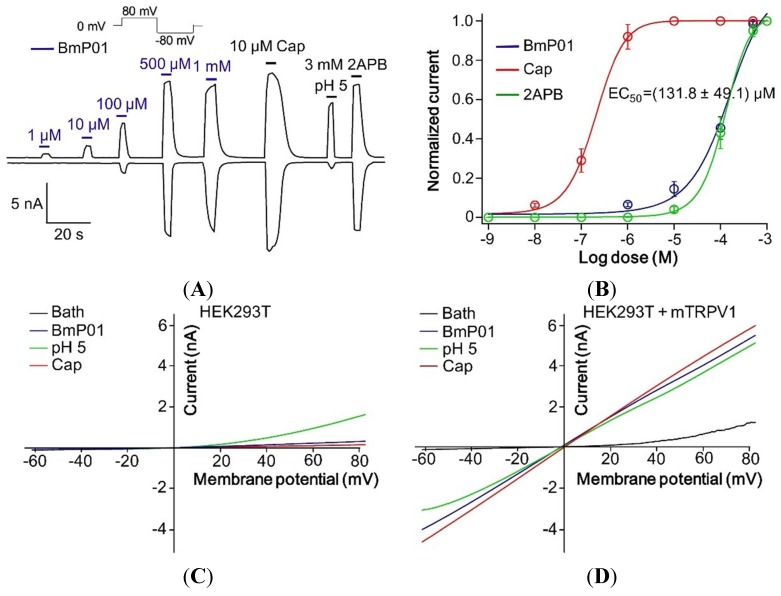
BmP01 activated TRPV1 channel. (**A**) Application of BmP01 at the concentrations of 1 µM, 10 µM, 100 µM, 500 µM and 1 mM showed the increasing activity with higher concentration to activate TRPV1 currents. Along with Capsaicin, proton and 2APB, 500 µM of BmP01 completely activates the TRPV1 channel; (**B**) Concentration—response yielded an EC_50_ of 131.8 ± 49.1 µM BmP01 (*n* = 10); (**C**) In HEK293T cells, negative for TRPV1 channel, BmP01 and capsaicin did not evoke the currents, whereas only proton induced some currents; (**D**) In contrast, BmP01 and capsaicin evoked the currents in HEK293T cells with over-expressed TRPV1 channels.

According to the findings from DRG neurons and animal behavior tests, BmP01 displayed a similar pharmacological response with capsaicin, the well-known agonist of TRPV1. We were interested in the investigation of the molecular interaction between BmP01 and TRPV1. Thus, electrophysiological tests on transient transfected TRPV1 were performed. According to the recordings from transient transfected mTRPV1 expressed on HEK293T cells, in electrophysiology, BmP01 with the concentration of 1 µM, 10 µM, 100 µM, 500 µM and 1 mM was applied and found to activate the mTRPV1 channel in a dose dependent manner. Five hundred micromoles of BmP01 along with capsaicin, proton and 2APB potently activated TRPV1 (Figure 5A). Determination of concentration–response relationship yielded an EC_50_ of 131.8 ± 49.1 µM on TRPV1 channel currents (Figure 5B). To our knowledge, HEK293T cells have been reported to endogenously express acid-sensing ion channels (ASICs) [39]. In order to check whether BmP01-induced currents were caused by the activation of ASICs, ramp method was used for comparing the toxin effects between TRPV1-expressed and native HEK293T cells. In native HEK293T cells, BmP01 as well as capsaicin did not evoke currents, but proton evoked current to some extent due to the endogenous ASICs (Figure 5C). Because of the expression level of endogenous ASICs, the currents evoked by proton hardly influenced the whole-cell currents in TRPV1-expressed cells. Thus, the same experiment carried out on TRPV1-expressed cells determined that BmP01-induced currents were elicited from TRPV1 channels (Figure 5D). These observations reveal that BmP01 acts as the agonist of mTRPV1 and consequently induces pain in the similar molecular pathway of capsaicin.

## 3. Discussion

The venom system of poisonous animals has developed sophisticated chemical strategies to capture prey and defend themselves from predators and competitors [40]. Scorpions are distributed worldwide as a kind of aggressive predators and their venoms evolve multifunctional peptide toxins. Unfortunately, although scorpion envenomation is known to produce pain, up to this moment, the sophisticated strategy of pain-inducing scorpion toxins is still unclear. Our work provides isolation and biological activities of a pain-producing molecule from scorpion venom. By screening protein fractions for pain inducing activity, a hydrophilic protein fraction was found with high pain-producing bioactivity (Figure 1B). By further purification and cDNA analysis, this pain-producing peptide toxin was determined as BmP01 (Figure 1C, D, Figure 2). According to the previous report [9], we checked the function of BmP01 on mKv1.1 and mKv1.3 by electrophysiology. Interestingly, no significant change was observed on the mKv1.1 currents in the presence of 1 mM BmP01 (Figure 3A). Comparing to the high affinity to rat Kv1.1, the variants of Kv1.1 in mouse may protect itself from the binding of scorpion toxins. This is an additional indication to understand the evolution in murine ion channels [21]. In order to investigate the evolutionary strategies of ion channels between rat and mouse, our further research will focus on the binding sites of BmP01 onto rKv1.1.

There are a few publications suggesting that voltage-gated sodium channel (VGSCs) related scorpion toxins produce pain in mammals [15,16,21,22,41]. There is a list of nociceptors for various types of pain. Importantly, most C-fiber nociceptors also respond to noxious chemical stimuli, such as acid or capsaicin, the pungent ingredient in hot chili peppers [42]. Peptide toxins from poisonous animals may act as “ligands” to evoke ligand-gated ion channels and produce painful feelings [43]. Scorpion venoms are well-known pain-producing chemical punch. To our knowledge, lack of agonist of ligand-gated ion channels has been found in scorpion venoms and the sophisticated chemical strategies of scorpion venom may have not been revealed thoroughly. Our study determined that BmP01 is a pain-producing peptide toxin and subsequently revealed that BmP01-induced pain is caused by TRPV1 activation in sensory neurons (Figure 4 and Figure 5). These results do not only suggest BmP01 is a multifunctional bio-toxin interacting with potassium channels and TRPV1, but also introduces the first toxin from scorpion venom that acts as a ligand to activate ligand-gated ion channel and produce pain. Multifunctional peptides designed by venomous animals, which are very ingenious, are generally used for preying, because, in the natural world, energy of predator is very precious for living. Efficient capture of poisonous animals seems to rely on the high affinity and multifunction of toxins. The mechanism of chemical defense is exquisite and complex. Bioactive peptides are found to be multifunctional in many cases. For instance, antimicrobial peptides from the secretion of frog skin also act as proteinase inhibitors and neurotoxins [44]. The toxins from spider can target TRPA1 and voltage-gated potassium channels as well [45].

Being small predators, scorpions pack distinct toxins in their venoms, which are evolved specifically for hunting and self-defense. Some toxins are lethal to insects and worms that form their major food supply, by acting on Nav channels [46]. Others inhibit vertebrate Kv channels to cause hyper-excitability [47,48]. These toxins resemble most known animal toxins. BmP01 is unique as it is a quick activator of mammalian nociceptor. The resultant burning pain has a clear defensive value. BmP01 adopts a different channel-binding strategy from the structurally distinct DkTx, a 75-amino acid large peptide toxin. DkTx binds TRPV1 slowly but reaches high affinity due to antibody-like binding of two active knots [31]. BmP01 binds much faster to achieve a high affinity to TRPV1 for inducing pain. These properties make BmP01 a powerful tool for elucidating the gating mechanisms of TRPV1. Our study reveals that BmP01 potently induces pain *in vivo* in WT mice, not in TRPPV1 KO mice. But in electrophysiology, the EC_50_ of BmP01 to activate TRPV1 channel is higher and that might be due to the heat activation threshold of TRPV1, as the experimental temperature (~20 °C) was much lower than mammal’s body temperature (37–39 °C). Here, we hypothesize that BmP01 may activate the TRPV1 with lower EC_50_ value at body temperature, as TRPV1 is a heat sensitive receptor. Moreover, this study indicates that BmP01 may influence the heat activation pathway of TRPV1 channel.

As an attractive target for pain medication, TRPV1 is currently being investigated as an entrance portal for pain-killer QX-314 to inhibit sensory neurons [49,50]. Peptide toxins such as conotoxins have showed great promises as a new type of pain medicine [6,51]. BmP01 and its derivatives, being synthesizable small peptide agonists, may open a novel path to directly control the activity of nociceptors. However, further studies are needed for better understanding on the mechanism of action of BmP01.

## 4. Experimental Section

### 4.1. Venom Collection

Adult scorpions (*Mesobuthus martensii*) (both sexes, *n* = 5000) were purchased from Shandong Province of China. Crude venoms were collected manually by stimulating the venom glands in the telson of scorpions using a 6 V alternating current. One milliliter of venom was mixed with 20 μL proteinase inhibitor cocktail (Sigma, P8340-5, St. Louis, MO, USA). Following collection, venoms were stored at −80 °C until further use. The scorpions were fed on worms and carried out in the plastic cages with open tops for easy monitoring. Each milking was carried out 2 weeks after the previous milking.

### 4.2. Peptide Purification

The crude venom (0.8 mL) was diluted with 3.2 mL 0.1 M phosphate buffered saline (PBS, pH 6.0) and centrifuged at 12000 rpm for 10 min. The supernatant was then applied to a Sephadex G-50 (Superfine, Amersham Biosciences, 2.6 × 100 cm) gel filtration column equilibrated with 0.1 M PBS. Elution was performed with the same buffer and fractions were collected in tubes each containing 3.0 mL. The absorbance of elutes was monitored at 280 nm to obtain the gel filtration profile. According to the gel filtration profile obtained from the absorbance of elutes monitored at 280 nm, five major peaks were collected and lyophilized, re-suspended in 5 mL of 0.1 M PBS, and purified further using reverse-phase high-performance liquid chromatography (RP-HPLC; Gemini C_18_ column, 5 μm particle size, 110 Å pore size, 250 × 4.6 mm) with linear gradient of 5%–100% solution B (99.9% acetonitrile, 0.1% TFA) over 95 min with a flow rate of 1.5 mL/min. The elution was monitored at 280 nm and all the eluted peptides were collected and lyophilized separately. The effect of lyophilized peptides was then checked on pain behavior in mouse model and the peptide having desired function was further purified fully on an analytical C_18_ RP-HPLC column.

### 4.3. Mass Spectrometric Analysis and Sequencing of Peptide

The molecular mass of purified peptide was determined by matrix-assisted laser desorption/ionization time-of-flight (MALDI-TOF) mass spectrometry. The amino acid sequence determination of purified peptide was performed by automatic Edman degradation in Applied ProciseTM 491-A protein sequencer (Shimadzu Corporation, Kyoto, Japan), which determines *N*-terminal sequence of the peptide. Afterward, a (BLAST) (NCBI) search was carried out to check the sequence similarity with other previously reported peptides.

4.4. cDNA Library Construction and Cloning the Gene Encoding BmP01

cDNA was synthesized as previously described [24]. Total RNA was extracted from the venom glands of scorpions 24 h following venom extraction using TRIzol (Life Technologies, Carlsbad, CA, USA) and used to prepare cDNA using a SMART™ PCR cDNA synthesis kit (Clontech Laboratories, Inc., Mountain View, CA, USA) . The cDNA was prepared using total RNA extracted from *Mesobuthus martensii* venom gland and served as the template to amplify the gene that encodes target peptide. PCR was done with degenerate primers designed from a combination of peptide sequence (direct sequencing of peptide, Edman degradation to amplify cDNA encoding BmP01. Following amplification, the fragment was sequenced. 

### 4.5. Cell Preparation

DRG neurons from WT or TRPV1 KO mice were acutely dissociated and maintained in short-term primary culture as previously described [52]. DRGs were incubated in 5% CO_2_/95% air at 37 °C for 1–4 h prior to patch-clamp experiments. HEK293T cells were used for mKv1.1 or mKv1.3 expression. Cells were grown under standard tissue culture conditions (5% CO_2_, 37 °C) in DMEM supplemented with 10% fetal bovine serum. Cells were transfected with DNA mixture (channel constructs and a green fluorescent protein (GFP) reporter plasmid) using lipofectamine 2000 transfection reagent (Invitrogen, Carlsbad, CA, USA) following the manufacturer’s instructions and used for electrophysiology 24–48 h after transfection. Cells with GFP fluorescence were selected for patch-clamp recordings 36–72 h after transfection.

### 4.6. Electrophysiological Recordings

All patch clamp recordings were carried out at temperature 20 °C using an EPC-10 amplifier (HEKA Electronik, Lambrecht, Germany) according to the previous report [52]. Recording pipettes were made from borosilicate glass capillary tubing, and their resistances were 3–5 MΩ when filled with internal solution. For mKv1.1 and mKv1.3 current recordings, the cells were held at −80 mV for 5 min after the whole-cell construction was established. All of the potassium current traces were elicited by a 300-ms depolarizing potential of 0 mV from a holding potential of −80 mV. The standard pipette solution contained (in mM): 140 KF, 4 MgCl_2_, 5 ATP-Na, 1 EGTA, and 10 HEPES, pH 7.3. The standard bath solution contained (in mM): 137 NaCl, 5.9 KCl, 2.2 CaCl_2_, 1.2 MgCl_2_, 14 glucose, and 10 HEPES, pH 7.3. For mice TRPV1 channel recordings, the pipette and bathing solution contained (in mM): 130 NaCl, 0.2 EDTA, and 3 HEPES, pH 7.2. Dose-response curves were fitted using the following Hill logistic equation:

*y* = 1 – (1 – *f*_max_)/[1 + ([*T_x_*]/EC_50_)*^n^*] (1)

where *n* is an empirical Hill coefficient and f_max_ is the fraction of current resistant to inhibition at high toxin (*T*_x_) concentration. τ_on_ and τ_off_ values were obtained from single exponential fits using the equations:

*I*(*t*) = *a*_0_ + *a*_1_[1 – exp(–*t*/τ_on_)] (2)

*I*(t) = *a*_0_ + *a*_1_ exp(–*t*/τ_off_) (3)

### 4.7. Animals

Mice, C57BL/6J, were used as wild type (WT) in these experiments. TRPV1-KO mice were purchased from Model Animal Research Center of Nanjing University, China. All the experiments using animals were carried out in accordance with recommendations in the Guide for the Care and Use of Laboratory Animals of Kunming Institute of Zoology, Chinese Academy of Sciences. Experimental protocols using animals in this work were approved by the Institutional Animal Care and Use Committees at Kunming Institute of Zoology, Chinese Academy of Sciences (2014-204). All efforts were made to reduce the number of animals used and to minimize the suffering of animals. 

### 4.8. Paw Licking

Following adaptation to the environment for 30 min, mice were injected with 10 μL of each drug with appropriate concentrations (kaliotoxin, capsaicin, BmP01, crude venom) at the plantar surface of the left hind paw (*n* = 10). Control mice were injected with the same volume of saline. Immediately after injection, the time of paw licking was recorded by digital video camera for 30 min.

### 4.9. Data Analysis

Experimental data of electrophysiological recordings were acquired and analyzed by using the Pulse program and Igor Pro. The data obtained in experiments involving animals were analyzed by GraphPad Prism 5 (GraphPad Software, Inc., San Diego, CA, USA). All of the data points are shown as the means ± S.E. *n* is presented as the number of experiments. Statistical analysis was carried out by Student’s *t* test, and * indicated a significant difference (*p* < 0.01).

## 5. Conclusions

The study on scorpion venom was carried out and anticipated to find a pain-inducing molecule and to clarify the molecular mechanism of it. Here we report a novel function of BmP01 from the venom of the scorpion *Mesobuthus martensii*. So far, to the best of our knowledge, this is the first report unveiling molecular pathway of a molecule from scorpion venom that induces pain by activating the TRPV1 channel. In our study, we discovered the molecular mechanism of a peptide, BmP01, which has previously been reported as an inhibitor of voltage gated (Kv) channel subtypes. However, interestingly, we found this peptide has the function of inducing pain in mice. The results of our study reveal that the scorpion uses BmP01 to target the TRPV1 channel for producing pain. We believe that the identification of pain-inducing compound may assist in finding a way to treat the pain induced by scorpion envenomation.
